# Comparative Genomics and Identification of an Enterotoxin-Bearing Pathogenicity Island, SEPI-1/SECI-1, in *Staphylococcus epidermidis* Pathogenic Strains

**DOI:** 10.3390/toxins10030093

**Published:** 2018-02-25

**Authors:** Xavier Argemi, Chimène Nanoukon, Dissou Affolabi, Daniel Keller, Yves Hansmann, Philippe Riegel, Lamine Baba-Moussa, Gilles Prévost

**Affiliations:** 1 Service des Maladies Infectieuses et Tropicales, Hôpitaux Universitaires, Strasbourg 67000, France; yves.hansmann@chru-strasbourg.fr; 2 EA 7290, Virulence Bactérienne précoce, Université de Strasbourg, CHRU Strasbourg, Fédération de Médecine Translationnelle de Strasbourg, Strasbourg 67000, France; dkeller@unistra.fr (D.K.); philippe.riegel@chru-strasbourg.fr (P.R.); prevost@unistra.fr (G.P.); 3 Faculté des Sciences et Techniques, Laboratoire de Biologie et de Typage Moléculaire en Microbiologie, Université d’Abomey, Calavi, Cotonou 05 BP 1604, Benin; chimnanouk85@yahoo.fr (C.N.); affobali_dissou@yahoo.fr (D.A.); laminesaid@yahoo.fr (L.B.-M.); 4 Laboratoire de Microbiologie du Centre National Hospitalier et Universitaire Hubert Koutoukou-Maga, Cotonou, 05 BP 1604, Benin

**Keywords:** pathogenicity island, enterotoxin, *Staphylococcus epidermidis*, *Staphylococcus aureus*, pan-genome, core genome

## Abstract

*Staphylococcus epidermidis* is a leading cause of nosocomial infections, majorly resistant to beta-lactam antibiotics, and may transfer several mobile genetic elements among the members of its own species, as well as to *Staphylococcus aureus*; however, a genetic exchange from *S. aureus* to *S. epidermidis* remains controversial. We recently identified two pathogenic clinical strains of *S. epidermidis* that produce a staphylococcal enterotoxin C3-like (SEC) similar to that by *S. aureus* pathogenicity islands. This study aimed to determine the genetic environment of the SEC-coding sequence and to identify the mobile genetic elements. Whole-genome sequencing and annotation of the *S. epidermidis* strains were performed using Illumina technology and a bioinformatics pipeline for assembly, which provided evidence that the SEC-coding sequences were located in a composite pathogenicity island that was previously described in the *S. epidermidis* strain FRI909, called SePI-1/SeCI-1, with 83.8–89.7% nucleotide similarity. Various other plasmids were identified, particularly p_3_95 and p_4_95, which carry antibiotic resistance genes (*hsrA* and *dfrG*, respectively), and share homologies with SAP085A and pUSA04-2-SUR11, two plasmids described in *S. aureus*. Eventually, one complete prophage was identified, ΦSE90, sharing 30 out of 52 coding sequences with the *Acinetobacter* phage vB_AbaM_IME200. Thus, the SePI-1/SeCI-1 pathogenicity island was identified in two pathogenic strains of *S. epidermidis* that produced a SEC enterotoxin causing septic shock. These findings suggest the existence of in vivo genetic exchange from *S. aureus* to *S. epidermidis*.

## 1. Introduction

*Staphylococcus epidermidis* is a coagulase-negative staphylococcus (CoNS) that forms a part of the commensal skin flora; however, it also acts as a major opportunistic pathogen causing nosocomial infections in hospital settings, primarily foreign body-associated infections, such as catheter-related bacteremia. The pathogenesis of *S. epidermidis* is related to the production of several adhesion factors and biofilm, coupled with a wide range of antibiotic resistance genes. Strong evidence supports the hypothesis that *S. epidermidis* can transfer mobile genetic elements (MGEs) to *Staphylococcus aureus* in vivo [1,2]. Until recently, the absence of toxin genes from *S. aureus* in the *S. epidermidis* genomes led researchers to postulate that *S. epidermidis* could not acquire genes from *S. aureus* in vivo, despite the evidence of horizontal gene transfer from *S. aureus* to CoNS and *Listeria* in vitro in the form of pathogenicity islands [3,4]. In 2011, Madhusoodanan et al. identified the *S. epidermidis* pathogenicity island SePI-1 in a clinical strain, *S. epidermidis* FRI909, which was isolated in the United States [5]. This pathogenicity island carried two enterotoxin genes, *sec3* and *sell*, which appeared functional, thereby offering the first evidence that the genetic materials could be transferred from *S. aureus* to *S. epidermidis*. In 2016, we described two strains of *S. epidermidis*, SE90 and SE95, in two patients with bacteremia and septic shock, and reported the production of staphylococcal enterotoxin C (SEC) and the presence of a coding sequence with 100% homology with *sec3*, as described in SePI-1 [6]. Sequence alignment indicated that *sec3* in SePI-1 was 95% identical to *sec3* from *S. aureus*. Madhusoodanan et al. also identified a chromosomal insertion sequence, named SeCI-1, which formed a composite genomic island with SePI-1 that lacked several genes found in *S. aureus* pathogenicity islands and was identified as a module participating in DNA packaging and replication. The probability of the recent transmission of this genomic island was consequently low, and, as expected, no evidence of mobilization capability for the sequence was found, despite the presence of the putative helper phage, φ909. In a large screening study of 200 strains isolated from bacteremia patients, no SePI-1 or other SePI-like genes were found, suggesting that the presence of the pathogenicity island in *S. epidermidis* is rare, or even unique [5]. The recent identification of a functional *sec3* sequence in the clinically-virulent *S. epidermidis* strains led us to hypothesize the presence of an underlying pathogenicity island. To better understand the genetic environments of this enterotoxin and to identify any other virulence factors with coding sequences located on a pathogenicity island, plasmid, or prophage, all putative MGEs from *S. epidermidis* strains SE90 and SE95, were sequenced. Furthermore, whole genome sequences of *S. epidermidis* strains and SEC-producing *S. aureus* strains in the GenBank database were compared.

## 2. Results

### 2.1. SE90 and SE95 Whole Genome Assembly and Annotation

The whole genome sequences of *S. epidermidis* strains SE90 and SE95 are presented in Table 1. Illumina paired-end read assemblies with SPAdes software produced 26 contigs for SE90 and 38 for SE95, with N90 = 39 kb and N90 = 43 kb, respectively. The ABACAS contig ordering tool and IMAGE gap closing with a scaffolding step produced two 2.4-Mb genomes, with GC contents between 31.95% and 32.47%. SE90 and SE95 comprised 2189 coding sequences, 1 tmRNA, 57–59 tRNAs, and 9–7 rRNAs. The sequences and annotations of the SE90 and SE95 genomes and their plasmids were deposited in the National Center for Biotechnology Information (NCBI) database under the accession numbers listed in Table 1 and Table 2.

### 2.2. Identification of a Sec3-Containing Pathogenicity Island

**Figure 1:**
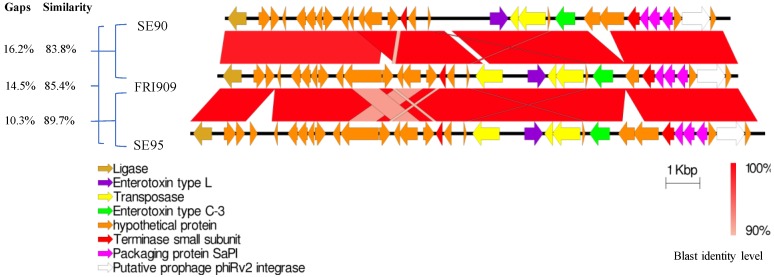
Nucleotide sequence alignments of the composite genomic island SePI-1/SeCI-1 from *S. epidermidis* FRI909 with similar regions from *S. epidermidis* SE90 and SE95 (Easyfig 2.2.2 for generating BLAST alignment files and visualization).

The IslandViewer 4 web server identified several putative genomic islands, the most significant finding being the genetic region with the *Sec3*-coding sequence. Using ARTEMIS, we manually improved the annotation around this gene using the Uniprot and BLAST databases. We identified a 20-kb-coding sequence that corresponded to the composite structure of SePI-1/SeCI-1, as described previously in *S. epidermidis* FRI909, with sequence homologies that ranged from 83.8% to 89.7%, according to the pairwise alignment using EMBOSS Needle from the EBI website. Using Easyfig, we aligned and visualized SePI-1/SeCI-1 along with the corresponding sequences from SE90 and SE95, and found that the overall gene predictions were similar, as was the organization (Figure 1). The genome of this genomic island was inserted downstream of the SsrA-binding protein, at the location of a unique tmRNA in this genome. As described in *S. epidermidis* FRI909, there were two sets of direct repeat sequences, DR1 and DR2, that flanked the two elements of the composite genomic island SePI-1/SeCI-1. As observed by Madhusoodanan et al., several transposases were located within the sequence, suggesting that this composite region resulted from several recombination events [5]. Multiple sequence alignment using Clustal Omega found a 100% amino acid sequence homology between these transposases [7].

### 2.3. Identification of Plasmids and Prophages

Several plasmids were identified within the nonaligned contigs that displayed an unexpected high coverage level after whole genome assembly. We identified two plasmids in the genome of SE90, p_1_90 and p_2_90, and four plasmids in the genome of SE95, p_1_95 to p_4_95, as detailed in Table 2. Only one plasmid, p_2_95, comprised replication and mobilization genes, and was, therefore, categorized as mobilizable. Two plasmids, p_1_90 and p_3_95, were nonmobilizable, but displayed a replication protein. The other three plasmids p_2_90, p_1_95, and p_4_95 were considered doubtful because they lacked any replication gene. These three plasmids, along with p_3_95, comprised antibiotic resistance genes, and a majority of their sequences matched those of the previously-described plasmids. Interestingly, p_3_95, a nonmobilizable plasmid, was nearly identical to SAP085A, a cyclin resistance plasmid identified in *S. aureus* (NCBI accession number GQ900437.1). No toxin-coding sequences or putative adhesion factors were identified.

**Figure 2:**
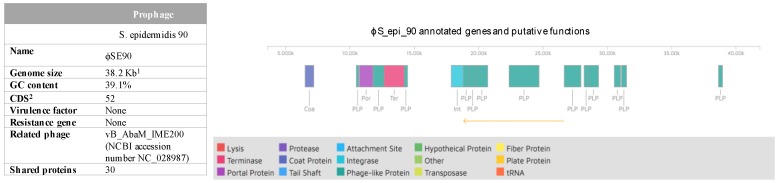
Prophage identification after whole genome sequencing of *S. epidermidis* SE90 and SE95 (using the Phaster web server). ^1^ Kb, kilobase; ^2^ CDS, number of coding sequences.

A search of prophage sequences within the main chromosomes and nonaligned contigs of SE90 and SE95 revealed only one intact prophage in SE90 and none in SE95. The SE90 phage was named ϕS_epi_90 and comprised no virulence or antibiotic resistance genes. It was a 38.2-kb sequence with 52 coding sequences and a GC content of 39.1%. These traits and its modular organization are characteristic of the *Siphoviridae* phage according to Kwan et al. (see Figure 2) [8]. It shared 30 out of 52 proteins with the *Acinetobacter* phage νB_Aba_IME200 (NCBI accession number NC_028987). No known virulence-associated genes were identified in this prophage.

### 2.4. Whole Genome Comparisons between S. epidermidis and S. aureus

**Figure 3:**
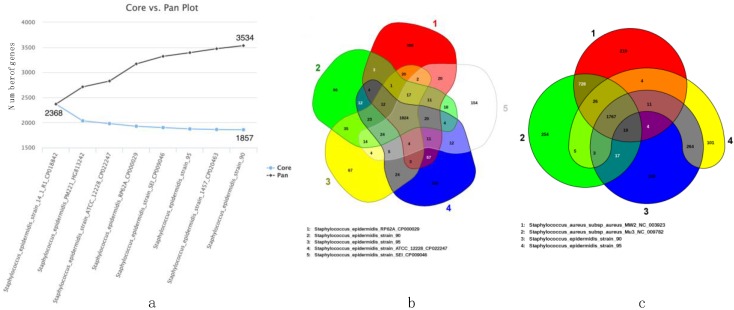
Core vs. pan genome development plot (panel **a**) and subsets (panel **b** and **c**) from *S. epidermidis* SE90 and SE95, and referenced *S. epidermidis* and *S. aureus* genomes from the NCBI genome server (EDGAR 2.2 software platform).

Nucleic acid identities, with previously complete published genomes, revealed 97–99% sequence homologies with *S. epidermidis* strains, and 76.5% with *S. aureus* strains Mu3 and MW2 (see Supplementary Material, Figure S1). Core versus pan genome development analysis of eight *S. epidermidis* genomes, including SE90 and SE95, revealed that 1857 genes formed the core genome of *S. epidermidis* and 3534 genes formed the pan genome (Figure 3a). Although the pan genome appeared to constantly grow, the core genome was limited to less than 2000 genes. The Venn diagram in Figure 3b,c, according to the EDGAR interface, indicated 66 and 67 singleton genes in SE90 and SE95, respectively. The order of the Venn diagram was limited to five genomes because the number of regions within the diagram of n^th^ order is 2^n^ − 1, which results in areas too small for graphical representation of more than five genomes. In addition, the singletons that corresponded to the genes without a reciprocal best hit to another genome as orthologs are not necessarily proper singletons.

**Figure 4:**
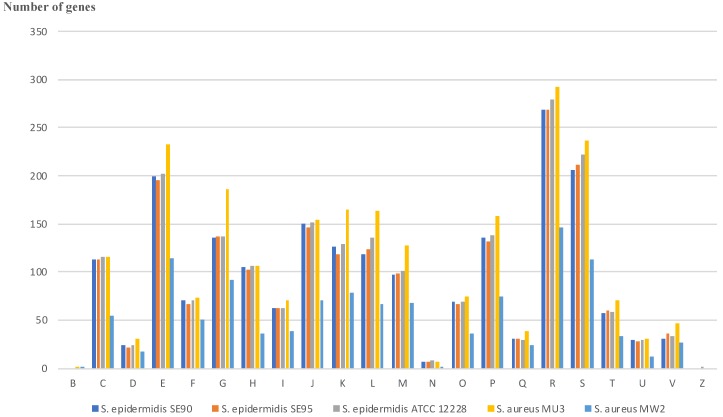
Clusters of orthologous groups (COG) of proteins from *S. epidermidis* SE90 and SE95 after whole genome annotations, and comparison with *S. aureus* MU3 (NCBI accession number NC_009782), *S. aureus* MW2 (NCBI accession number NC_003923), and *S. epidermidis* ATCC12228 (NCBI accession number CP022247.1) (webMGA software platform with an *e*-value cutoff for prediction = 0.001). All detailed results are available in Supplementary Materials, Table S1. COG categories are as follows: For cellular processes and signaling, D is cell cycle control, cell division, and chromosome partitioning; M is cell wall/membrane/envelope biogenesis; N is cell motility; O is posttranslational modification, protein turnover, and chaperones; T is signal transduction mechanisms; U is intracellular trafficking, secretion, and vesicular transport; V is defense mechanisms; and Z is cytoskeleton. For information storage and processing, B is chromatin structure and dynamics; J is translation, ribosomal structure, and biogenesis; K is transcription; and L is replication, recombination, and repair. For metabolism, C is energy production and conversion; E is amino acid transport and metabolism; F is nucleotide transport and metabolism; G is carbohydrate transport and metabolism; H is coenzyme transport and metabolism; I is lipid transport and metabolism; P is inorganic ion transport and metabolism; and Q is secondary metabolites biosynthesis, transport, and catabolism. R is for general function prediction only, and S is for unknown function.

Therefore, we used the dedicated interface in EDGAR to identify singletons, excluding orthologs, and confirmed that the *sec3* and *sell* genes were unique to SE90 and SE95. The second element of interest is the identification of a type III-A CRISPR/Cas system in SE95 singletons, similar to that of *S. epidermidis* RP62A [9,10]. The Venn diagram in Figure 3c indicates that SE90, SE95, and the *S. aureus* strains have 1767 genes within their core genomes, and only 100 and 101 singletons for SE90 and SE95, respectively. A majority of the genes that distinguished SE90 and SE95 from both *S. aureus* strains are involved in the metabolic pathways. The function of each gene from the *S. epidermidis* strains, as well as that from *S. aureus* Mu3 and MW2, were classified into COG categories, and the results are shown in Figure 4. The diagram was limited to four genomes for better graphical representation; however, all results are available in the Supplementary Materials (Table S1). Most genes did not fall into a specific COG category (unknown or general function), and genes for amino acid transport and metabolism were the most abundant. We found that the partition based on COG categories was similar between *S. epidermidis* strains; however, it distinctly differed from that of *S. aureus*.

## 3. Discussion

The characterized presence of superantigens in CoNS is extremely rare. We characterized two clinical strains from two patients, including one who died from septic shock at 48 h after diagnosis of *S. epidermidis* bacteremia. We found no definitive evidence of a causative link between the production of SEC3 and the clinical course of the patient; however, an association seemed probable. Indeed, toxic shock syndromes are known to originate from the streptococcal and *S. aureus* superantigens, including SEC [11]. Among CoNS, *S. epidermidis* is presumably the most studied species because it produces several virulence factors, such as adhesion factors, biofilm, and phenol-soluble modulins, which are likely involved in clinical manifestations, such as endocarditis, osteomyelitis, and material associated infections [12,13,14].

We identified an enterotoxin-coding sequence in a composite genomic island previously described in *S. epidermidis* FRI909, with high nucleotide similarity and similar coding sequence. This genomic island, SePI-1/SeCI-1, presumably resulted from a genetic exchange from *S. aureus* to *S. epidermidis*. Our findings suggest that this was not an isolated event, even though Madhusoodanan et al. did not succeed in mobilizing the genomic island. This enterotoxin-bearing pathogenicity island could be transmissible between *S. epidermidis* strains; however, as a second hypothesis, it might also result from a repeated and organized exchange of SaPIs from *S. aureus* to *S. epidermidis*, employing mechanisms that remain to be elucidated. It has been suggested that the common presence of a Type III-A CRISPR system in *S. epidermidis* could interfere with the horizontal genetic transfer and prevent the acquisition of foreign DNA from different species. Nevertheless, we identified CRISPR-coding sequences in SE95 singletons, with similar sequences to those described in *S. epidermidis* RP62A, although SE95 may have acquired SePI-1/SeCI-1 from *S. aureus*. Another hypothesis states that the genomic island was acquired from another *S. epidermidis* strain; however, as evidenced by Madhusoodanan et al., it seems nonmobilizable while integrated in the *S. epidermidis* genome [5]. Identification of MGEs in SE90 and SE95 led us to characterize three particular plasmids, p_1_95, p_3_95, and p_4_95, which were previously described in *S. aureus* strains, suggesting the existence of genetic exchanges between these two species. In addition, the comparative genomic analyses between *S. aureus* and *S. epidermidis* emphasize their genetic proximity, even if the presence of several CRISPR genes and the existence of restriction-modification systems in *S. aureus* might prevent horizontal gene exchanges [15]. Our study is limited by the number of *S. aureus* genomes that were included, knowing that 202 complete genomes are presently available according to GenBank. Comparative genomic studies could certainly address these interrogations; however, they would require that all complete genomes from the compared species are available.

Eventually, sequence analysis revealed constant growth in the pan genome, whereas the core genome rapidly decays and stagnates below 2000 genes. In a recent study of the pan and core genomes of 30 clinical strains of *S. epidermidis*, Conlan et al. reported high diversity between the strains, even from a single individual, a particularity that relies on the open pan genome of *S. epidermidis* [16]. Similar to our study, the authors found that the core genome size fits an exponential decay curve that plateaus at 1960 genes, whereas the pan genome fits a power law curve. Post et al. performed comparative genomic analyses among *S. epidermidis* strains from orthopedic device-associated infections [17] and found no clear correlation between the lineage and clinical outcomes; however, they observed a strong association with the biofilm formation capacity and antibiotic resistance. Thus, the comparative genomics studies provide a powerful and innovative tool to explore the genome-wide associations in staphylococci as several methods, software, and genomes become available [18].

## 4. Conclusions

In conclusion, by using whole genome sequencing, we identified two clinical and virulent *S. epidermidis* strains that caused septic shock and produced enterotoxin C with a coding sequence located on a pathogenicity island. The structure of this pathogenicity island suggests that it might have originated from *S. aureus*, as its description in another *S. epidermidis* strain in the literature also suggested that this is not an isolated event. At present, the variety and the extent of such genetic transfers remain unclear.

## 5. Materials and Methods

### 5.1. Bacterial Strains, Culture, and DNA Extraction

The two strains, SE90 and SE95, were previously isolated from two children with bacteremia at the NTH-HKM Hospital, Cotonou, Benin [6]. The strains were cultured on Columbia agar with 5% sheep blood and incubated for 24 h at 37 °C. Bacteria were identified at the species level using matrix-assisted laser desorption/ionization time-of-flight mass spectrometry, according to the manufacturer’s instructions (Bruker Daltonics, Wissembourg, France). DNA was extracted using the MasterPure^TM^ DNA Purification Kit (Epicentre, Le Perray-en-Yvelines, France). DNA purity was controlled through optical density (OD), with OD 260/280 ≥ 1.8 and OD 260/230 ≥ 1.9. DNA was dissolved in RNAse-, DNAse-, and protease-free 10 mM Tris–HCl buffer (pH 8–8.5). The visual quality of the extracted DNA was assessed using agarose gel electrophoresis and UV visualization to exclude DNA degradation or contamination.

### 5.2. Genome Sequencing, Assembly, and Annotations

Whole genome sequencing was performed as previously described using Illumina technology (Illumina HiSeq 2500; GATC Biotech AG, Konstanz, Germany) [19]. Sequencing produced paired-end sequences of 125 bp. Sequence assemblies were identified using SPAdes (v 3.10.1) (Algorithmic Biology Laboratory, St Petersburg Academic University, St Petersburg, Russia) with 21-33-55-77-97 k-mers. SPAdes output contigs of < 1000 bp or with coverage of <10× were removed. Sequences were completed using the post-assembly genome-improvement toolkit from the Sanger Institute [20]. The final sequence annotation was performed with the NCBI genome annotation pipeline. The unique chromosomes for each sequence and short, nonaligned contigs were further analyzed to identify the plasmids or other genetic elements of interest. Nonaligned contigs were loaded into the ARTEMIS software (v.16.0.0) (Wellcome Trust Sanger Institute, Hinxton, Canbridge, United Kingdom) to identify the open reading frames (ORFs), which were successively analyzed using the UniProt database.

### 5.3. Identification of Pathogenicity Islands, Plasmids Coding Sequences, and Prophages

MGEs were explored as previously described [19]. Pathogenicity islands were identified using IslandViewer 4 [21], which associates IslandPick, SIGI-HMM, and IslandPath-DIMOB for the identification of pathogenicity islands. This tool displays the results as circular graphical images and allows localization of the putative pathogenicity islands using gene coordinates. Prophage search and annotation were performed using PHage Search Tool Enhanced Release [22], which allows the rapid identification of putative prophage sequences and provides annotations. A quality score of >90 indicates an intact prophage sequence. Plasmid search was performed on all nonaligned contigs that remained after the genome assembly, in particular, those that displayed an unusually high level of coverage that would be possibly linked to sequence duplication in the bacterial genome. Those additional contigs were annotated using the NCBI annotation pipeline. Plasmid categorization was performed according to the terminology used by Smillie et al., which distinguishes the mobilizable plasmids from conjugative ones depending on the presence of a Type IV secretion system (T4SS) [23]. The presence of a relaxase gene, but not a T4SS, corresponded to a mobilizable plasmid, although a helper plasmid might be needed to assemble the conjugation system. The presence of a relaxase gene and T4SS genes corresponded to a conjugative plasmid. A plasmid without relaxase and T4SS was considered as nonmobilizable, and if the replication coding sequence was also absent, the plasmid was considered doubtful. Indeed, considering the methodology used to find plasmids (extra chromosomal contigs with unexpectedly high coverage) the presence of a *rep* gene would be expected. Consequently, its absence made the plasmid identification doubtful. All coding sequences from identified MGEs were loaded separately into the ARTEMIS software and characterized using the UniProt database to search for putative virulence factors (toxins, adhesion proteins, and immune evasion factors) [13,14,24,25,26].

### 5.4. Identification and Comparative Genomic of the SEC3-Coding Sequence

IslandViewer allowed the identification of a pathogenicity island that comprised the SEC3 coding sequence, which was aligned to the *S. epidermidis* FRI909 pathogenicity island using EMBOSS Needle from the EBI website to evaluate nucleotide and amino acid identities. Easyfig (v.2.2.2) was used to generate a BLAST alignment file with SePI-1/SeCI-1 from *S. epidermidis* FRI909, with a minimum length of 100 bp, maximum e-value of 0.001, and minimum identity value of 90 [27].

### 5.5. Whole-Genome Sequence Analysis

SE90 and SE95 whole genome sequences were compared with all other complete *S. epidermidis* genomes. The NCBI Genome database presently contains six complete and 388 draft sequences [28]. ATCC12228 was used as the reference strain (novel sequence publicly available in 2017 using PacBio sequencing technology; NCBI accession number CP022247.1). The five other complete *S. epidermidis* genomes that were included in the analysis were: PM221 (NCBI accession number HG813242), SEI (NCBI accession number CP009046), RP62A (NCBI accession number CP000029.1), 14.1.R1 (NCBI accession number CP018842.1), and 1457 (NCBI accession number CP020463.1). Two *S. aureus* complete genomes were also included in the analysis: MU3 (NCBI accession number NC_009782) and MW2 (NCBI accession number NC_003923). *S. epidermidis* ATCC12228 (deposited by FDA, USA) is a nonbiofilm forming, noninfection-associated strain. *S. aureus* Mu3 and MW2 both contain the enterotoxin genes *sec3* and *sei*, located on the pathogenicity island SaPImw2 of strain MW2. Draft genomes, such as for *S. epidermidis* FRI909 (NCBI accession number GCA_000186205.2, a draft genome with 69 contigs greater than 500 bp, ranging from 558 bases to 293 kb), were omitted from the whole genome comparisons. Indeed, the draft genomes comprise an unordered set of contigs that cannot be used for proper whole genome comparisons. Identification of the core and pan genomes, and phylogeny analyses were performed using the EDGAR software platform (v.2.2) (Bioinformatics and Systems Biology, Julius-Liebig-University Giessen, Hesse, Germany) [29]. This web tool performs analogy analyses with a cut-off value that automatically adjusts according to the input data. The ortholog analysis was based on the BLAST score ratio values to predict the pan, core, and accessory genomes [18]. For EDGAR phylogeny analyses, as specified in the manual, the pipeline uses the core genome, and every set of the orthologous genes found in all genomes is separately aligned using the multiple alignment tool MUSCLE [30]. Alignments are concatenated and used to calculate a distance matrix. EDGAR also allows the calculation of gene subsets (core genome, singletons) between different species such as *S. epidermidis* and *S. aureus*. Functional analyses of the putative proteins encoded by the SE90 and SE95 genomes, were compared with all other strains using the Clusters of Orthologous Groups of proteins (COGs) database. COG categories were retrieved using the WebMGA software platform (Center for Research in Biological Systems, University of California San Diego, California, USA), with an e-value cut-off of 0.001 for prediction [31,32].

## Figures and Tables

**Table 1:** Whole genome sequencing of *Staphylococcus epidermidis* strains SE90 and SE95, in comparison with the *Staphylococcus epidermidis* reference strain ATCC 12228.

*S. epidermidis* Strain	ATCC 12228 ^2^	*S. epidermidis* SE90	*S. epidermidis* SE95
NCBI accession number	CP022247.1	CP024408	CP024437
Clinical origin	Not clinical	Bacteremia	Bacteremia
N50 after SPAdes assembly (kb) ^1^	-	179	195
N90 after SPAdes assembly (kb)	-	39	43
Contigs number after SPAdes assembly ^3^	-	26	38
Mean coverage after SPAdes assembly	-	422×	410×
Contigs number after PAGIT assembly termination	-	7	10
Full length of the scaffold (bp) ^4^	2,497,508	2,395,274	2,407,125
GC content (%)	32.03	32.47	31.95
Coding sequences	2545	2189	2189
tRNA ^5^	60	57	59
rRNA ^6^	19	9	7
tmRNA ^7^	0	1	1

^1^ Kb, kilobase; ^2^ Sequence obtained with long-sequence reading technology: PacBio sequencing technology; ^3^ SPAdes output: coverage cutoff 10× and length cutoff 1000 bp; ^4^ bp, base pairs; ^5^ tRNA, transfer RNA; ^6^ rRNA, ribosomal RNA; ^7^ tmRNA, transfer-messenger RNA.

**Table 2:** Plasmid identification after whole genome sequencing of *S. epidermidis,* SE90 and SE95.

	Plasmids					
	*S. epidermidis* SE90		*S. epidermidis* SE95			
Name	p_1_90	p_2_90	p_1_95	p_2_95	p_3_95	p_4_95
Accession number	CP024409	CP024410	CP024438	CP024439	CP024440	CP024441
Genome size ^1^	18.6 kb	6.6 kb	12.1 kb	9 kb	4.5 kb	3.3 kb
Contig coverage	1968×	2095×	1212×	1214×	3040×	1567×
GC content	26.12%	28.17%	28.69%	29.29%	30.29%	33.56%
CDS ^2^	20	7	14	10	4	3
Virulence factor	None	None	None	None	None	None
Resistance gene	None	Beta-lactamase	Beta-lactamase	None	*hsrA* ^4^	*dfrG* ^5^
Replication gene	*repA*	None	None	*repA*	*repN*	None
Mobilization module	None	None	None	MobA	None	None
T4CP/T4SS genes	None	None	None	None	None	None
Plasmid category	Non-mobilizable	Doubtful	Doubtful	Mobilizable	Non-mobilizable	Doubtful
Related plasmid (strain, nucleotide length, CDS)	p1457 (*S. epidermidis*, 17 CDS, 15 kb)	pSC-SNUDS-2-1 (*S. cohnii*, 30 CDS, 29.4 kb)	pETB DNA (*S. aureus*, 63 CDS, 60.5 kb)	pVISLISI_5 (*S. lugdunensis*, 13 CDS, 12.6 kb)	SAP085A (*S. aureus*, 3 CDS, 4.4 kb)	pUSA04-2-SUR11 (*S. aureus*, 28 CDS, 26 kb)
Nucleotide similarities:						
- Sequence cov ^3^	−42%	93%	75%	52%	100%	88%
- Identities	99%	97%	99%	91%	99%	99%
- E-value	0.0	0.0	0.0	0.0	0%	0.0

^1^ kb, kilobase; ^2^ CDS, number of coding sequences; ^3^ Sequence cov, coverage of the sequence identified in *S. epidermidis* 90 and 95 with the closet related plasmid (according to BLAST). ^4^ Cycline resistance; ^5^ Trimethoprim-resistant dihydrofolate reductase.

## Supplementary Materials

The following are available online at http://www.mdpi.com/2072-6651/10/3/93/s1: Figure S1. Mean nucleic acid identity between the whole genome sequences of SE90 and SE95 from this study, and six *S. epidermidis*, and two *S. aureus* sequences available from the NCBI genome server (EDGAR 2.2 software platform); Table S1. Clusters of orthologous groups (COG) of proteins from complete published *S. epidermidis* genomes and comparisons with *S. aureus* MU3 and MW2 (webMGA software platform with an e-value cutoff for prediction = 0.001).

## Appendix

The sequence and annotations of SE90 and SE95 genomes and their plasmids have been deposited in the NCBI database under the accession numbers:

- SE90: CP024408
- Plasmid p1_90: CP024409
- Plasmid p2_90: CP024410
- SE95: CP024437
- Plasmid p1_95: CP024438
- Plasmid p2_95: CP024439
- Plasmid p3_95: CP024440
- Plasmid p4_95: CP024441
