# Supplementary material for: Comparative Genomics and Identification of an Enterotoxin-Bearing Pathogenicity Island, SEPI-1/SECI-1, in Staphylococcus epidermidis Pathogenic Strains
Source: Toxins (Basel). 2018 Feb 25;10(3):93. doi: 10.3390/toxins10030093 (PMC5869381; doi:10.3390/toxins10030093)
Supplement: Supplementary file 1 [file toxins-10-00093-s001.pdf]

Supplementary Materials: Comparative Genomics and Identification of an Enterotoxin-Bearing Pathogenicity Island, SEPI-1/SECI-1, in *Staphylococcus epidermidis* Pathogenic Strains

Xavier Argemi, Chimène Nanoukon, Dissou Affolabi, Daniel Keller, Yves Hansmann, Philippe Riegel, Lamine Baba-Moussa and Gilles Prévost

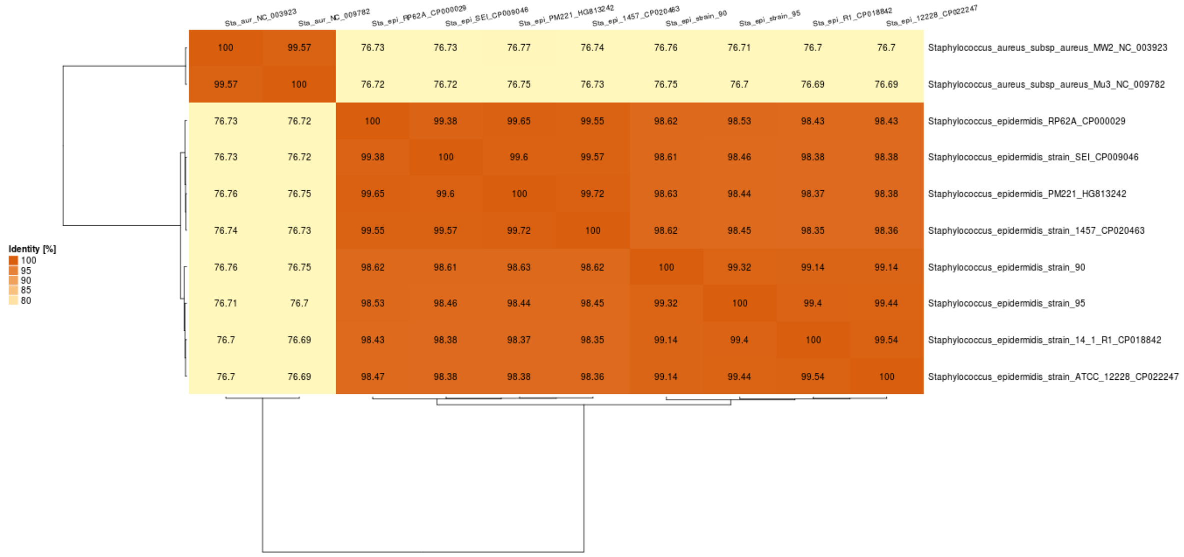

Figure S1. Mean nucleic acid identity between whole genome sequences of SE90 and SE95 from this study, six S. epidermidis, and two S. aureus sequences available from NCBI genome server (EDGAR 2.2 software platform).

Table S1: Clusters of Orthologous Groups (COG) of proteins from *Staphylococcus epidermidis* complete published genomes, and comparison with *Staphylococcus aureus* MU3 and MW2 (webMGA software plateform with an e-value cutoff for prediction =0.001).

COG description	COG class	*S. epidermidis* SE90	*S. epidermidis* SE95	*S. epidermidis* ATCC 12228	*S. aureus* MU3	*S. aureus* MW2	*S. epidermidis* RP62A	*S. epidermidis* 14-1-R1	*S. epidermidis* 1457	*S. epidermidis* SEI	*S. epidermidis* PM221
Chromatin structure and dynamics	B				1	1					
Energy production and conversion	C	113	113	116	116	55	116	67	67	113	55
Cell cycle control, cell division, chromosome partitioning	D	24	21	24	31	17	24	9	8	23	13
Amino acid transport and metabolism	E	200	196	202	233	114	200	104	109	208	113
Nucleotide transport and metabolism	F	70	67	70	73	50	76	24	25	71	39
Carbohydrate transport and metabolism	G	136	137	137	186	92	142	88	78	140	61
Coenzyme transport and metabolism	H	105	103	106	107	36	109	68	67	105	39
Lipid transport and metabolism	I	63	63	63	71	38	65	29	30	61	32
Translation, ribosomal structure and biogenesis	J	150	146	151	154	70	156	79	82	152	59
Transcription	K	126	118	129	165	78	132	71	71	128	60
Replication, recombination and repair	L	118	123	135	164	66	169	67	68	146	69
Cell wall/membrane/envelope biogenesis	M	97	98	101	128	68	102	57	56	102	47
Cell motility	N	7	7	8	7	2	7	5	5	7	3
Posttranslational modification, protein turnover, chaperones	O	69	67	69	75	36	72	36	36	70	31
Inorganic ion transport and metabolism	P	135	132	138	158	75	142	74	79	140	60
Secondary metabolites biosynthesis, transport and catabolism	Q	30	31	29	38	24	31	15	16	30	20
General function prediction only	R	269	269	279	292	146	281	148	142	278	132
Function unknown	S	206	211	222	237	113	219	126	121	210	93
Signal transduction mechanisms	T	57	60	58	70	33	67	34	38	61	30
Intracellular trafficking, secretion, and vesicular transport	U	29	28	29	31	12	28	16	14	29	13
Defense mechanisms	V	31	36	33	46	27	36	20	21	31	16
Cytoskeleton	Z			1				1			
